# Feasibility of Laparoscopic Removal of the Largest Documented Uterine Fibroid Without Morcellation

**DOI:** 10.3390/reports8020071

**Published:** 2025-05-17

**Authors:** Jacek J. Sznurkowski, Jakub Wnuk

**Affiliations:** 1 Profesor Jacek Sznurkowski Podmiot Leczniczy, ul. Stefana Żeromskiego 23A, 81-246 Gdynia, Poland; jakwnuk@gmail.com; 2 LuxMed Szpital Gdańsk S.A., ul. Wileńska 44, 80-215 Gdańsk, Poland

**Keywords:** fibroid, myomectomy, subserosal fibroid, laparoscopy, uterine preservation, morcellation, trocar placement, large fibroids, left upper quadrant entry, LUQ, direct trocar entry, DTE, fragmentation

## Abstract

**Background and Clinical Significance**: Uterine fibroids affect up to 25% of women of reproductive age and can lead to significant symptoms or impact fertility, often requiring surgical management. While hysteroscopic myomectomy is suitable for intracavitary fibroids, *intramural* and subserosal fibroids typically necessitate open or minimally invasive surgery (MIS). Laparoscopic approaches offer notable advantages, including reduced postoperative pain and faster recovery. However, MIS is frequently avoided in cases of very large fibroids due to technical difficulty and concerns about safe tissue extraction. Power morcellation, previously used for specimen removal, has fallen out of favor due to the risk of disseminating occult malignancies, especially in women over 35. Therefore, establishing the **feasibility of MIS without morcellation** in such cases is essential. **Case Presentation**: A woman of reproductive age presented with a symptomatic uterine fibroid measuring approximately 4 kg (1500 cm^3^). Laparoscopic myomectomy was performed using a modified trocar entry technique and contained tissue fragmentation, avoiding morcellation. The operation was completed successfully without complications. Postoperative recovery was uneventful, and the patient was discharged on postoperative day two. Histopathological examination confirmed a benign leiomyoma. **Conclusions**: This case highlights the **feasibility of laparoscopic removal** of an exceptionally large uterine fibroid without morcellation. Through careful patient selection, strategic trocar placement, and controlled tissue fragmentation, MIS can be safely performed in select high-volume cases. These findings support reconsidering the size limitations of laparoscopic myomectomy when conducted by experienced surgeons using appropriate techniques.

## 1. Background

Uterine fibroids affect approximately 25% of women of reproductive age [1,2]. In appropriately selected patients, myomectomy may be indicated not only for those seeking to preserve fertility but also for women experiencing significant fibroid-related symptoms [2,3].

Fibroids are commonly categorized by their location within the uterus: intracavitary, intramural, and subserosal. Hysteroscopic myomectomy remains the first-line conservative surgical approach for symptomatic intracavitary fibroids. For women with intramural fibroids who wish to conceive, ablative procedures such as uterine artery embolization (UAE) are generally discouraged due to adverse effects on fertility [4,5]. A randomized controlled trial comparing UAE with myomectomy demonstrated lower pregnancy rates and higher miscarriage rates in the UAE group [4].

As a result, intramural and subserosal fibroids often require surgical excision to manage symptoms and maintain reproductive potential [3,6]. The two principal surgical options for removing these fibroids are open surgery and minimally invasive surgery (MIS), which includes laparoscopic or robot-assisted approaches. The choice of technique depends on various factors, including the surgeon’s proficiency—particularly in laparoscopic suturing—the anticipated operative time based on fibroid size and number, and the relative cost, with laparoscopic surgery typically being more expensive.

A key challenge in laparoscopic or robot-assisted myomectomy is the removal of excised tissue. Power morcellation, once widely adopted, is now discouraged due to the risk of disseminating occult leiomyosarcoma, particularly in women over 35 years of age—the group most commonly affected by fibroids [7,8,9]. Therefore, the development of alternative, oncologically safe tissue extraction techniques in MIS is essential.

This report describes what is likely the largest uterine fibroid (4 kg/1583 cm^3^) ever removed laparoscopically without morcellation. The case highlights two main contributions: first, it demonstrates the feasibility of MIS even in cases of extreme fibroid size; second, it outlines critical surgical elements—including patient selection, trocar placement, and tissue fragmentation strategies—that enabled safe and effective laparoscopic myomectomy in this setting.

## 2. Case Description

A 38-year-old nulliparous woman presented for the evaluation of a large, symptomatic uterine fibroid. The fibroid had been slowly enlarging over several years. Her primary symptoms were lower abdominal pain, constant pressure on the bladder and progressively worsening anemia.

Approximately five months prior to presentation, she had been evaluated by a gynecologist who recommended open surgery with hysterectomy. Due to a strong desire to preserve fertility, she declined the proposed treatment and sought care at a center with extensive experience in laparoscopic myomectomy. She ultimately qualified for minimally invasive surgery at the outpatient clinic of LuxMed Hospital S.A. in Gdańsk, Poland, and admitted to the Gynecology Department three months later.

The patient underwent a two-month course of preoperative treatment with Ryego, which was discontinued due to intolerable menopausal symptoms. Preoperative transvaginal ultrasonography performed five months before surgery and again on the day of surgery by two different sonographers indicated stabilization of fibroid growth, with estimated volumes ranging from 1200 to 1391 cm^3^ and 1185 to 1300 cm^3^, respectively (Table 1).

The patient was a healthy, non-smoking woman with a slender build (177 cm height, 72 kg weight, BMI-23). Her most recent Pap smear was normal.

On physical examination, a large pelvic–abdominal mass was noted, extending approximately 12 cm above the umbilicus. Imaging with both magnetic resonance imaging (MRI) and ultrasound confirmed an 18 cm × 10 cm × 12 cm subserosal fibroid originating from the anterior uterine wall.

A preoperative hysteroscopy was not performed, as imaging showed a 1 cm distance between the fibroid and the endometrial cavity, with a normal ultrasound appearance of the endometrium.

The patient underwent laparoscopic myomectomy under general anesthesia with endotracheal intubation. The patient was positioned in dorsal lithotomy (33-degree step Trendelenburg), and a Foley catheter was inserted and left in place for 16 h.

**Figure 1:**
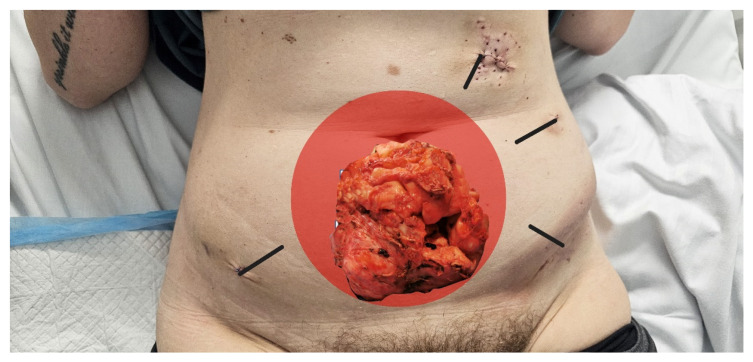
Trocar placement demonstrates alignment with the principle of a 60-degree angle between instruments.

Abdominal access was established using a left upper quadrant (LUQ) entry with a 12 mm incision 2 cm below the left costal margin. A 10 mm, 30-degree laparoscope was introduced to provide optimal visualization. Pneumoperitoneum was achieved and maintained at 12 mmHg. Two additional trocars (5 mm and 12 mm) were inserted on the left side of the abdomen along a virtual arc extending from the laparoscope port to a point 1 cm above the anterior superior iliac spine. The instruments were spaced approximately one hand width apart, allowing for a 60-degree angle between the camera and working instruments, ensuring optimal triangulation during surgery (trocar placement is depicted in Figure 1).

Intraoperatively, a large fibroid measuring 18 cm × 14 cm × 12 cm was identified, arising from the anterior uterine wall and displacing reproductive organs toward the sigmoid colon and exerting pressure on the urinary bladder. The fibroid extended into the anterior cul-de-sac and penetrated deeply into the right retroperitoneal space, where it reached the external iliac vessels and the right ureter. A second subserosal fibroid, located on the posterior wall of the uterus, measured 2 cm × 2 cm × 3 cm.

The ovaries, fallopian tubes, and remaining abdominal and pelvic organs were grossly normal.

The right ureter was carefully dissected free using cold technique. The bladder was mobilized from the fibroid. The main fibroid was excised using a harmonic scalpel, and its feeding vessels were coagulated with a 5 mm Maryland Ligasure (Covidien). The posterior fibroid was removed using a Sonic scalpel. Resulting myometrial defects were closed in multiple layers with individual absorbable sutures.

**Figure 2:**
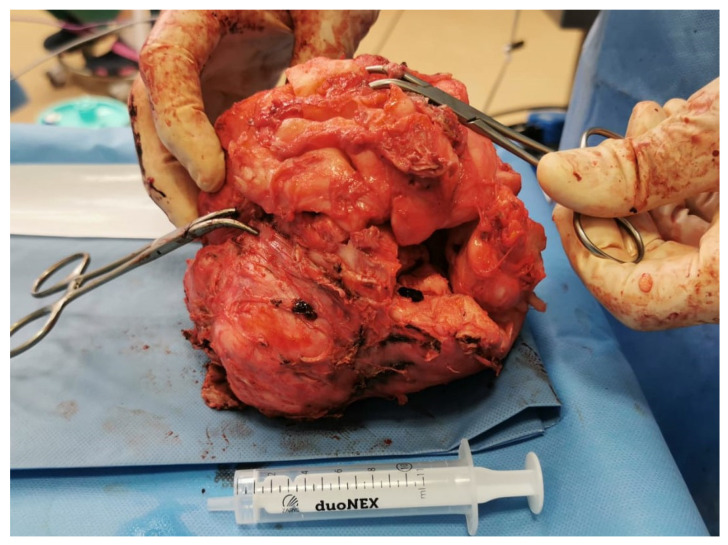
Ex vivo reconstruction of the fibroid: All removed fragments were reassembled to approximate the original dimensions (18 cm × 14 cm × 12 cm; 1583 cm^3^) and total weight of 3980 g.

The uterine cavity was reconstructed to its proper shape with absorbable sutures, and the serosa was closed with a continuous suture. Tissue extraction was performed without morcellation. The fibroids were manually fragmented into approximately 2 cm pieces using scissors and an L-hook, and removed through a 3 cm trocar site enlargement. The total volume of excised tissue was 1583 cm^3^, and the total weight was 3980 g (see Figure 2) All tissue was submitted for histopathological analysis, which later confirmed benign leiomyoma. A Redon drain was placed in the pelvic cavity. Hemostasis was confirmed. CO_2_ pneumoperitoneum was released, and the trocars were removed. The duration of the procedure was 6.5 h.

The patient was mobilized 12 h after the procedure (the following morning), beginning with one minute of standing in an upright position, followed by a brief one-minute walk within her room. Approximately one hour later, the urinary catheter was removed, and the patient was given a light liquid meal. Within the next few hours, the Redon drain was removed, and her diet was gradually advanced to include clear soup and a buttered roll. A postoperative blood test revealed a hemoglobin level of 10.5 g/dL, with all other parameters within normal limits.

Painkillers were administered at the patient’s request, and no intravenous fluids were given, as she was encouraged to maintain hydration by drinking still water.

The patient was discharged approximately 36 h after surgery in good general condition, independently mobile, tolerating oral intake, and without signs of complication. She was given written instructions for home care and scheduled for outpatient follow-up to assess wound healing and review histopathological results.

## 3. Discussion

Laparoscopic myomectomy for large uterine fibroids presents significant technical challenges, particularly due to restricted working space and limited instrument maneuverability. The rationale for this report was to demonstrate a reproducible surgical strategy that enables safe laparoscopic removal of an extremely large fibroid without morcellation, an oncologically controversial practice. In doing so, the authors aimed to contribute practical solutions to a procedure often considered contraindicated in similar scenarios.

The main finding of this study is that a fibroid weighing 3980 g and measuring 1583 cm^3^ can be safely and effectively removed laparoscopically without power morcellation. To the authors’ knowledge, this is the largest fibroid documented in the literature to be removed via laparoscopy using manual fragmentation and without any uterine manipulator. This case illustrates that minimally invasive surgery (MIS) remains feasible in carefully selected patients, even in cases of extreme uterine enlargement, and provides a replicable model of technique, trocar placement, and ergonomic strategy.

The technique described here builds upon existing knowledge by highlighting the importance of maintaining a 60-degree angle between instruments to optimize ergonomics, minimize collision, and facilitate effective dissection and suturing [10,11]. Strategic trocar positioning within a 15–20 cm arc from the target organ, which in this case was achieved via left upper quadrant (LUQ) entry at Palmer’s point, allowed for superior visualization and instrument control. Previous studies rarely report this approach for large fibroids, favoring umbilical access, which may compromise working angles and ergonomics [12,13,14,15].

Patient anthropometry was crucial in determining feasibility. The patient’s height (177 cm), pubis-to-xiphoid distance (36 cm), and interspinous distance (30 cm) provided the anatomical space necessary for safe laparoscopic manipulation of a massively enlarged uterus [10,11]. The absence of a vaginal uterine manipulator reduced the risk of uterine perforation and facilitated en bloc dissection. This approach may offer a safer alternative in complex cases where standard uterine manipulation is not feasible.

The case also underscores the risks associated with power morcellation. The literature reports serious complications, including bowel, bladder, and vascular injuries, as well as six deaths associated with its use between 1993 and 2013 [16]. The incidence of morcellator-related organ injury is estimated at 0.12% [17]. Furthermore, morcellation has been linked to dissemination of both benign fragments (parasitic leiomyomas) and malignant cells in cases of undiagnosed uterine sarcoma [18,19,20,21,22,23,24,25,26]. One in 350 women undergoing surgery for presumed fibroids may harbor occult uterine cancer [27], and the risk increases with age [28]. While in-bag morcellation has been proposed as a compromise, its effectiveness in preventing dissemination remains under investigation, and it can impair visibility [27].

As a result, major societies, including the Society of Gynecologic Oncology (SGO), American College of Obstetricians and Gynecologists (ACOG), and the U.S. Food and Drug Administration (FDA) have issued strong warnings or outright discouraged the use of laparoscopic power morcellation during hysterectomy or myomectomy [29,30,31]. The fragmentation method described in this case avoids these risks entirely and offers an oncologically safe alternative.

From a reproductive perspective, laparoscopic myomectomy offers significant benefits for women desiring future pregnancy. Studies have shown postoperative pregnancy rates exceeding 60% [32,33], and minimally invasive techniques are associated with better quality of life and improved sexual function compared to open surgery [34]. Furthermore, patients undergoing laparoscopic procedures typically experience faster recovery and return to normal activities [35]. In this case, the patient was discharged within 36 h and returned to work within 10 days, reflecting the accelerated convalescence achievable with MIS.

However, economic considerations can limit access to such procedures. The extended operative time (6.5 h) and use of advanced energy devices such as the harmonic scalpel and LigaSure contribute to increased costs. In many healthcare systems, reimbursement remains standardized regardless of complexity, making total abdominal hysterectomy (TAH) a more financially viable option for institutions. This case highlights the need to reassess reimbursement models to reflect the long-term societal and reproductive benefits of conservative, fertility-preserving treatments.

Strengths of this case include its detailed surgical description, novel trocar approach, and safe tissue removal strategy. Limitations include its single-case design, lack of long-term fertility follow-up, and the fact that the success of the procedure was dependent on the surgeon’s high level of laparoscopic expertise and favorable patient anatomy.

In summary, this case reinforces the feasibility of laparoscopic myomectomy for very large fibroids without morcellation, offering a technically sound, oncologically safe, and fertility-preserving option for selected patients.

## 4. Conclusions

This case confirms that laparoscopic myomectomy without morcellation is a viable and safe option, even for exceptionally large fibroids, when performed by skilled surgeons using strategic techniques. Challenges such as limited space, tissue extraction, and uterine repair can be overcome with proper planning and anatomical consideration. Fibroid size alone should not be viewed as a contraindication for MIS; instead, surgical outcomes are largely influenced by technical proficiency and patient body habitus. These findings support expanding MIS indications and highlight the need for further research into anatomical predictors of feasibility.

## Figures and Tables

**Table 1:** Fibroid volume measurements at preoperative time points: Five months before surgery and on the day of surgery. Note the slight discrepancy in assessments by two different ultrasonographers, though both confirm a volume greater than 1200 cm^3^).

Gynecologic TVUS Report	Time	2D Measurement	Volume
Swissmed Outpatient Clinic	5 months before the operation	18.05 cm × 11.4 cm × 12.91 cm	1391 cm^3^
LuxMed Gynecology Department	Day of surgery	18.1 cm × 10.33 cm × 12.04 cm	1205 cm^3^

## Data Availability

The original contributions presented in this study are included in the article. Further inquiries can be directed to the corresponding author.
